# The complete chloroplast genome of *Sibbaldianthe bifurca* Linnaeus

**DOI:** 10.1080/23802359.2021.1914219

**Published:** 2021-06-14

**Authors:** Min Wang, Pengcheng Lin, Yu Fan

**Affiliations:** a Key Laboratory for Qinghai-Tibet Plateau Phytochemistry of Qinghai Province, College of Pharmacy, Qinghai Nationalities University, Xining, P.R. China; b School of Chemistry and Chemical Engineering, Qinghai Nationalities University, Xining, P.R. China

**Keywords:** *Sibbaldianthe bifurca*, chloroplast genome, phylogenetic analysis

## Abstract

The complete chloroplast genome of *Sibbaldianthe bifurca* Linnaeus was sequenced, assembled and annotated. It is a circular form of 156,734 bp in length, which was separated into four distinct regions, a large single copy (LSC) of 83,450 bp, a small single-copy region (SSC) of 18,286 bp, two inverted repeats (IR) of 27,499 bp. After annotation, a total of 133 genes were predicted, of which, 87 encode proteins, eight rRNA, 38 tRNA. The evolutionary history, inferred using the Maximum Likelihood (ML) method, indicates that *S. bifurca* was grouped within Rosaceae, and comprised a clade with *Sibbaldianthe adpressa* with 100% Bootstrap value.

*Sibbaldianthe bifurca* Linnaeus, belonging to Rosaceae, is a sympodially growing long-rooted, nonrosette, polycarpic perennial herb with yellow flowers, and has a wide range of distribution, from European Russia, West Siberia, Central Asia Mongolia to China (Basargin 2007; Godin 2009). For containing some chemical constituents, such as flavonoid, this plant was used extensively in traditional medicines for curing ulceration, cancer, hepatitis, etc., and some bioactive constituents have been identified, such as quercetin-4′-O-beta-D-glucoside from methanol extracts of this plant. It inhibits tyrosinase strongly (Zhao et al. 2008; Piao et al. 2009; Tomczyk et al. 2010). Apart from researches on medicines, some literatures on the population structure and ontogeny have also been reported (Basargin 2007; Godin 2009). In this study, we report the complete chloroplast (cp) genome of *S. bifurca*.

Samples from Qilian mountains (36°34’37*"*N,101°48’27*"*E) in Qinghai Province were collected for sequencing which was performed on the Illumina Novaseq platform (Shenzhen Huitong biotechnology Co. Ltd). Voucher specimen (HCPQNU-20200531001) was deposited in the Herbarium, College of Pharmacy, Qinghai Nationalities University. A sample’s total genomic DNA was extracted from about 100 mg fresh leaves using a modified CTAB method (Murray and Thompson 1980). Paired-end Libraries with an average length of 350 bp were constructed and sequenced. The complete cp genome was assembled with the de novo assembler SPAdes (Bankevich et al. 2012) and annotated via PGA (Qu et al. 2019).

The complete cp genome of *S. bifurca* (GenBank accession no. MT796626.1) has a typical quadripartite form of 156,734 bp in length and composed of a large single-copy region (LSC, 83,450 bp), a small single-copy region (SSC, 18,286 bp), two inverted repeats (IR, 27,499 bp). GC content of the genome is 37.1%. A total of 133 genes were predicted on this cp genome, of which, 87 encode proteins, eight rRNA, and 38 tRNA.

Phylogenetic analysis was performed based on complete cp genomes of *S. bifurca* and other 31 related species reported in Rosaceae, three species in Leguminosae as out-group. The sequences were aligned using MAFFT (Katoh et al. 2002), and trimAl was employed to remove ambiguously aligned sites (Capella-Gutierrez et al. 2009). The evolutionary history was inferred with the Maximum Likelihood (ML) method using IQ-TREE 1.6.12 under GTR + F+R3 model (Nguyen et al. 2015; Kalyaanamoorthy et al. 2017), and the output file was edited in MEGA 7.0 (Kumar et al. 2016). Bootstrap (BS) values were calculated with UFBoot2 from 1000 replicates analysis (Hoang et al. 2017). As expected, *S. bifurca* was grouped within Rosaceae and comprised a clade with *Sibbaldianthe adpressa* with 100% BS value (Figure 1). The complete cp genome of *S. bifurca* will be helpful for further studies on population genetics, taxonomy or resources protection.

**Figure 1.**
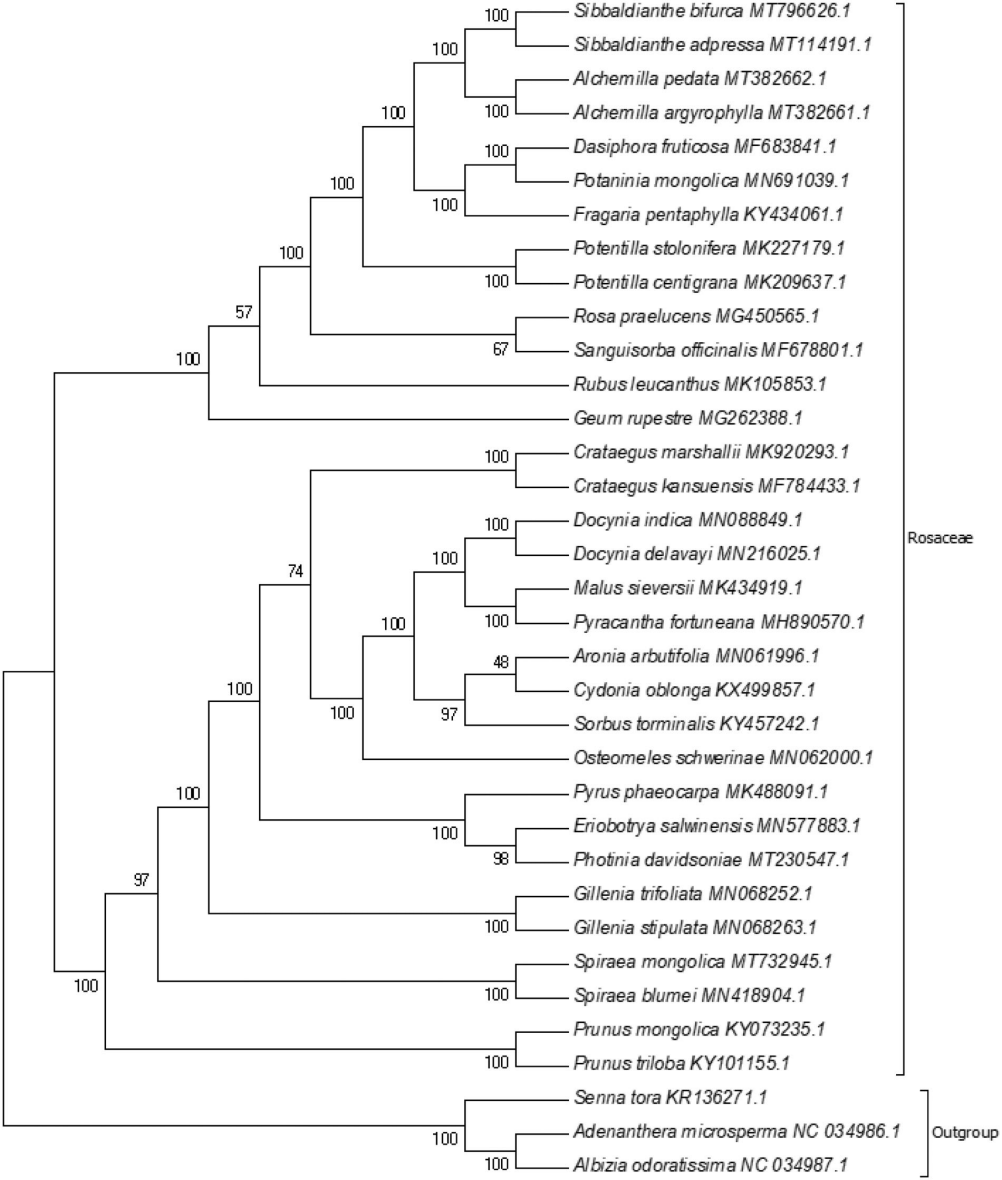
ML phylogenetic tree based on 35 species chloroplast genomes was constructed using IQ-TREE 1.6.12. Numbers on each node are bootstrap from 1000 replicates.

## Data Availability

The genome sequence data that support the findings of this study are openly available in GenBank of NCBI at (https://www.ncbi.nlm.nih.gov/nuccore/MT796626.1) under the accession no. MT796626.1. The associated BioProject, SRA, and Bio-Sample numbers are PRJNA678812, SRR13070755, and SAMN16813076, respectively.
